# Live-Imaging Analysis of Target Vessels and Nitric Oxide Production Associated with Gosha-Jinki-Gan and Keishi-Bukuryo-Gan: Two Herbal Preparations with Clinically Proven Blood Flow-Improving Effects but with Different Traditional Clinical Indicative Patterns

**DOI:** 10.1155/2022/3821345

**Published:** 2022-05-11

**Authors:** Aki Hirayama, Tsutomu Tomita, Takashi Nishida, Yumiko Nagano

**Affiliations:** ^1^Center for Integrative Medicine, Tsukuba University of Technology, 4-12-7 Kasuga, Tsukuba, Ibaraki 3058521, Japan; ^2^Timelapse Vision Inc., 5-23-11 Honcho, Shiki, Saitama 3530004, Japan

## Abstract

Gosha-jinki-gan (GJG) and Keishi-bukuryo-gan (KBG) are Kampo traditional herbal prescriptions used for different clinical patterns (*sho*) that improve blood flow. The pharmacological basis of the therapeutic choice remains unclear, although the clinical reliance of this pattern-based therapy is widely proven. We aimed to investigate their effects on microcirculation and nitric oxide (NO) kinetics using a live-imaging system to provide evidence for this. Live-imaging was performed in murine subcutaneous vessels and rat mesentery. In the subcutaneous vessels, we analyzed the effects of both drugs on the vessel diameter, blood flow velocity, and volume in the arteries, arterioles, and capillaries. In the rat mesentery, we induced the “*oketsu*” blood stasis using a stack of thin vinylidene chloride films and examined the effect on NO production using a fluorescent diaminofluorescein-2 diacetate. Following dissolution in hot water, 300 mg/kg of both drugs were administered intragastrically via a transesophageal catheter. Live-imaging analysis of subcutaneous blood flow revealed the different effects of GJG and KBG on their target vessels and effect onset. GJG targeted the capillaries and progressively increased the blood flow velocity and rate at 30–120 min after administration. No vasodilation or increased blood flow in the arteries and arterioles occurred. In contrast, KBG increased the diameter of the arterioles and arteries at 30–90 min after administration, and increased blood flow velocity and rate in arteries and arterioles. In a model of *oketsu* blood stasis in the mesenteric arteries, KBG increased the NO production from the vascular endothelial cells with dilatation of the arteriolar diameter. GJG improved blood flow mainly in the capillaries. Endothelial NO production decreased after GJG administration. The empirical treatment choice between GJG and KBG is based on the difference in target vessels and NO action and provides a pharmacological basis for therapy based on traditional medicine.

## 1. Introduction

Herbal medicine has been recently accepted worldwide as a complementary and alternative therapy to modern Western medicine. Kampo, a traditional Japanese herbal medicine, was originally introduced to Japan from ancient China and has developed by adapting to the unique vegetation and social customs of Japan. Currently, Kampo is an officially approved medical system that has demonstrated beneficial treatment outcomes and reduced side effects [1, 2]. The pharmacological action of Kampo formulae includes its effects on oxidative or nitrosative stress, which are recognized as one of the major therapeutic mechanisms [3–7].

In Kampo medicine, therapeutic strategies are determined according to “*sho*,” a clinical indicative pattern, which was diagnosed based on the principles of Kampo [8]. *Sho* is the traditional system used in East Asia; however, the concept differs partially between Kampo and traditional Chinese medicine [8]. One of the key concepts of *sho* diagnosis is the equilibrium between two symmetrical statuses, namely, Yin-Yo and Kyo-Jitsu (deficiency and excess, respectively). These equilibria are translated to the concepts of modern medicine as homeostasis, including acid-base and oxidative-reductive/antioxidative balance. Despite the diagnosis being similar to the Western medicine, the drug of choice differs for dissimilar *sho*. The clinical efficacy of such traditional medical treatments has been widely demonstrated despite the fact that the pharmacological mechanisms by which differences in *sho* affect the therapeutic effects remain unclear.

This study aimed to reveal these unknown pharmacological mechanisms and provide a modern medical background to traditional therapeutic theories. We analyzed the pharmacological effects of two Kampo formulae, namely, Gosha-jinki-gan (GJG) and Keishi-bukuryo-gan (KBG), which have similar clinical therapeutic targets but different *sho* indications, on microcirculation and nitric oxide (NO) production. Clinically, GJG and KBG effectively improve hemodynamics, but have different *sho* indications. GJG and KBG are both used to treat chillness or other symptoms caused by a circulatory disturbance in the lower extremities. However, GJG is also used to treat back pain and neuralgia and has recently been observed to reduce the side effects of chemotherapy, including the use of paclitaxel [9, 10]. KBG is widely used for “*oketsu*,” a traditional concept of circulation disturbance in Kampo, which is not restricted to the arterial area but extends to the venous system and capillaries. Both prescriptions are widely used for traditional treatment, but their therapeutic theories are based on the empirical treatment of humans. Moreover, the pharmacological basis, particularly in microcirculation, remains unclear [11–14].

We investigated the pharmacological effects on microcirculation by an *in vivo* live-imaging system, which evaluated changes in vessel diameter, erythrocyte blood flow velocity, and blood flow rate in three different types of vessels (i.e., arteries, arterioles, and capillaries). We aimed to employ the following two systems: murine subcutaneous vessels for physiological circulation and rat mesenteric vessels for a pathological *oketsu* circulation model [15]. The pharmacological effect on NO was investigated in the *oketsu* circulation model. Recent advances in circulation evaluation, such as Doppler ultrasonography, magnetic resonance imaging, and angiography, enable the analysis of the pharmacological effects on arterial and venous hemodynamics. In contrast, it is technically difficult to depict hemodynamics at the capillary level. In addition, NO and reactive oxygen species (ROS) play major roles in microcirculatory dynamics and are reportedly related to oxidative stress pathology [16].

## 2. Materials and Methods

### 2.1. Materials

Animals were purchased from Japan SLC (Hamamatsu, Japan). NO-detective fluorescent diaminofluorescein-2 diacetate (DAF-2DA) was obtained from Goryo Kayaku (Sapporo, Japan). N^G^-Monomethyl-L-arginine acetate (L-NMMA) was obtained from Dojindo Molecular Technologies, Inc. (Kumamoto, Japan).

The confocal laser scanning microscope imaging system consisted of an LSM 700 (ZEISS, Oberkochen, Germany) and ImageJ ver.1.45 s image analysis software (NIH, Bethesda, ML, USA). GJG (TJ-107) and KBG (TJ-25) were provided by Tsumura Co. Ltd. (Tokyo, Japan).

### 2.2. Experimental Design

We examined the physiological effects of GJG and KBG in murine subcutaneous blood vessels using 5-week-old male C57BL/6 mice. We evaluated the vessel diameter, erythrocyte blood flow velocity, and blood flow rate in the arteries, arterioles, and capillaries. For the *oketsu*-microcirculation disturbing model, we used the mesenterium of 8-week-old female Wistar rats according to our previous reports with minor modifications [15, 17]. Animal species and sex were selected primarily for their suitability for live imaging.

### 2.3. Kampo Formulae

GJG and KBG used in the investigation were clinical-grade dry extract granules. Both subcutaneous and mesenteric vessel studies evaluated the pharmacological effects of the GJG, KBG, and control groups. The therapeutic dose of GJG used in this study was 7.5 g/day, containing 1.75 g of the dry extract of mixed herbal crudes in the following proportions: *Rehmanniae radix* (5.0 g), *Achyranthis radix* (3.0 g), *Corni fructus* (3.0 g), *Dioscoreae rhizoma* (3.0 g), *Plantaginis semen* (3.0 g), *Alismatis rhizoma* (3.0 g), *Poria sclerotium* (3.0 g), *Moutan cortex* (3.0 g), *Cinnamomi cortex* (1.0 g), and *Aconiti radix* (1.0 g). Similarly, the therapeutic dose of KBG was 7.5 g/day, containing 3 g of each of the following crudes: *Cinnamomi cortex*, *Paeoniae radix*, *Semen persicae*, *Poria sclerotium*, and *Moutan corte*. The three-dimensional high-performance liquid chromatography (3D-HPLC) fingerprints of GJG and KBG produced by the same process as the products used in this study, provided by the manufacturer, are shown in Additional Material 1.

Each Kampo preparation was dissolved in hot water at 90°C, cooled to room temperature (23°C), and administered intragastrically via a gastric tube at a dose of 300 mg/kg at a concentration of 100 mg/mL. The control group received the same treatment and volume of saline solution.

### 2.4. Live-Imaging of Murine Subcutaneous Vessels

Live-imaging studies were performed at the laboratory of Timelapse Vision Inc. (Saitama, Japan) under the approval of the organization's ethics committee (approval number: 19_TUT_001, approval date: 2019/5/29). Experimental animals were euthanized in accordance with standard animal experimentation guidelines after the experiment was completed. We performed murine subcutaneous live-imaging under urethane anesthesia (1.5 g/kg, i.s.). Following an incision, the ventral skin of the mouse was peeled off, and the subcutaneous blood vessel was positioned on a glass plate. The vessel was covered with a thin vinylidene chloride film and fixed to prevent moisture evaporation. Subsequently, Kampo drugs or control saline was administered to the mice. Each group consisted of 3–4 mice.

The microcirculation of the subcutaneous blood vessels was recorded using real-time imaging before drug administration, at every 30 min up to a total of 120 min. We analyzed the inner vessel diameter, erythrocyte flow velocities, and blood flow volume in the arteries (diameter >50 *μ*m), arterioles (diameter, 10–50 *μ*m), and capillaries (diameter <10 *μ*m). The diameters of the blood vessels were measured in triplicate for each point on the images. We calculated the erythrocyte flow velocity by measuring the distance traveled by a single erythrocyte before and after a specific number of video frames, and dividing the number of frames by the elapsed time. This velocity was determined by performing quadruplicate measurements at a single point and was reported in the form of average values. The blood flow rate was calculated by assuming the cross-sectional area of the vessel to be a perfect circle and multiplying it by the blood flow velocity. The results of each measurement are presented as a percentage change from the initial values presented in Additional Material 2.

### 2.5. Live-Imaging of Microcirculation and NO Release in the Rat *Oketsu* Model

The *oketsu* model was created based on our previous report [15]. For the NO study, we used DAF-2DA as a specific fluorescent indicator. Fluorescence images were obtained by confocal laser scanning microscopy, with a wavelength of 488 nm for excitation and 492 nm for detection. As the fluorescence of DAF is accumulative, the local perfusion method was employed to eliminate its effect [15]. Following a peritoneal incision, we placed the mesentery on a glass plate and covered it with thin vinylidene chloride films to induce the *oketsu* status. A catheter was inserted into a vessel upstream of the set site to achieve a perfusion route. The observation sites of the upstream, midstream, and downstream portions of the perfusion area of a single mesenteric artery, which mainly reflected the arteries, arterioles, and capillaries, respectively, were determined under an intravital microscope.

To obtain a pre-drug administration fluorescence image, DAF-2DA (50 *μ*M) was administered via a fixed catheter and perfused locally for 3 min. At 30 min after this process, with the disappearance of fluorescence confirmed, Kampo drugs were administered intragastrically. At 60 min after administration, DAF-2DA was re-perfused for 3 min and a second round of images were obtained at the same site. In each imaging, fluorescence images were acquired in a total of six regions of interest, and the average fluorescence intensity was obtained using ImageJ. The change in fluorescence intensity was calculated by subtracting the baseline intensity from that of the second image.

To confirm the specificity of DAF-2 against NO, we measured the changes in fluorescence with the NO synthetase inhibitor L-NMMA. In particular, 25 mg/kg of L-NMMA was intravascularly administered 5 min before the second DAF-2DA perfusion, and a second dose of L-NMMA was added to the DAF-2DA solution at a final concentration of 2.5 mg/mL. The other process was performed in the same manner as in the previous measurement.

### 2.6. Statistical Analyses

Statistical analyses were performed using Prism 9 for macOS (GraphPad Software Inc., La Jolla, CA, USA). Two-way repeated measures analysis of variance (ANOVA) followed by Dunnett's multiple comparisons was employed for the time course analysis. The results are expressed as means ± standard errors of the mean (SEMs), except otherwise noted, and the *P* values for the multiple comparisons are indicated with symbols in the figures.

## 3. Results

### 3.1. KBG Dilated the Diameter of the Subcutaneous Arterioles and Arteries, While GJG Exerted No Effect on the Vessel Diameters

**Figure 1:**
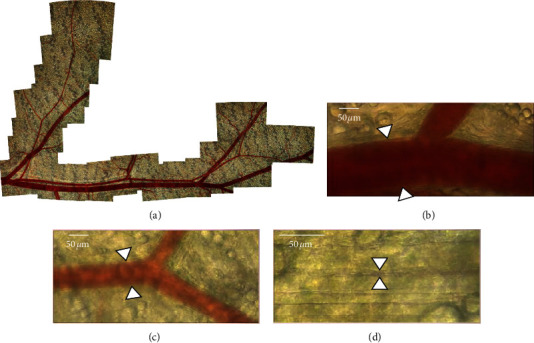
Typical analyzed region of murine subcutaneous vessels. Representative images of a typical analyzed murine subcutaneous region. (a) Low-magnification image of the analysis area perfused by a single artery; b, c, and d. Typical images of the analyzed artery (b), arteriole (c), and capillary (d). The imaging video files used for the analysis are provided in Additional Material 3 (GJG) and 4 (KBG).

Figure 1 depicts a typical subcutaneous vascular region observed by live imaging. In all vessels, blood flow was continuously observed without a thrombus or interruption of blood flow during the experiment. The measured values of the vessel diameter, red blood cell blood flow velocity, and blood flow rate in each group before drug administration are presented in Additional Material 2. There were no significant differences among the three groups before drug administration.

**Figure 2:**
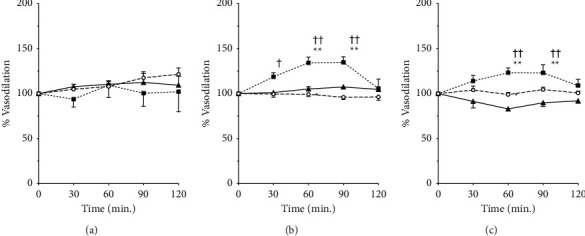
Vasodilative effects of Gosha-jinki-gan and Keishi-bukuryo-gan. Changes in the vasodiameter of murine subcutaneous capillaries (a), arterioles (b), and arteries (c) before and after the administration of Gosha-jinki-gan (circle, *n* = 4), Keishi-bukuryo-gan (square, *n* = 3), and control (triangle, *n* = 4). All panels are expressed as percentages and those before the administration as 100%. Each bar represents the mean ± SEM. ^*∗∗*^: *P* < 0.01 vs. control. ^††^: *P* < 0.01 vs. 0 min.

Figure 2 depicts the changes in the blood vessel diameter before and at 30–120 min after the administration of each Kampo preparation. The imaging video files used for the analysis are provided in Additional Materials 3 (GJG) and 4 (KBG). Additional materials can be found on Google drive (Google Corp., Mountain View, CA, USA) at the link provided in the Supplementary Materials section. There was no significant change in the capillary diameter (Figure 2(a)). KBG significantly dilated the diameter of the arterioles up to 135% of the pre-administration level and that of the arteries up to 123% between 60 and 90 min, post-administration, when compared to the control group (Figures 2(a) and 2(b)). There was no significant effect of GJG on the arterioles and arteries. The F- and *P* values of the two-way ANOVA were as follows: *F*(8,32) = 0.89 and *P*=0.5342 for capillaries, *F*(8,36) = 9.5 and *P* < 0.0001 for arterioles, and *F*(8,28) = 3.9 and *P*=0.0035 for arteries.

### 3.2. Increase in Subcutaneous Erythrocyte Blood Flow Velocity in Capillaries by GJG and in Arterioles by KBG

**Figure 3:**
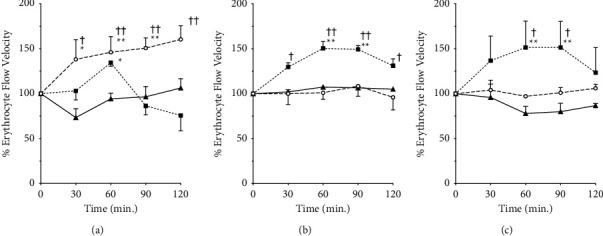
Effects of Gosha-jinki-gan and Keishi-bukuryo-gan on the erythrocyte flow velocity. Changes in the erythrocyte flow velocity of murine subcutaneous capillaries (a), arterioles (b), and arteries (c) before and after the administration of Gosha-jinki-gan (circle, *n* = 4), Keishi-bukuryo-gan (square, *n* = 3), and control (triangle, *n* = 3) are presented. All panels are expressed as percentages and those before administration as 100%. Each bar represents mean ± SEM. ^*∗*^: *P* < 0.05, ^*∗∗*^: *P* < 0.01 vs. control. ^†^: *P* < 0.05, ^††^: *P* < 0.01 vs. 0 min.

Figure 3 depicts the changes in erythrocyte blood flow velocity before and at 30–120 min after the administration of each Kampo preparation. GJG increased the capillary blood flow rate up to 160% of that before administration (Figure 3(a)). In contrast, KBG increased the erythrocyte blood flow velocity in arterioles up to 151% of pre-administration (Figure 3(b)). The effect of GJG on the erythrocyte blood flow velocity increased gradually up to 120 min after administration, while that of KBG reached a maximum at 60 min post-administration and began to decrease after 90 min. In the arteries, ANOVA did not reveal significant differences between the groups according to the drug and time course. The F- and *P* values of the two-way ANOVA were as follows: *F*(8, 32) = 3.3 and *P*=0.0076 for capillaries, *F*(8,28) = 4.0 and *P*=0.0031 for arterioles, and *F*(8,24) = 2.3 and *P*=0.0593 for arteries. The measured values of the red blood cell velocity in each group before drug administration are provided in Additional Material 2. The imaging video files used for the analysis are provided in Additional Materials 3 (GJG) and 4 (KBG).

### 3.3. Subcutaneous Blood Flow Volume Increased in Capillaries by GJG and in Arterioles and Arteries by KBG

**Figure 4:**
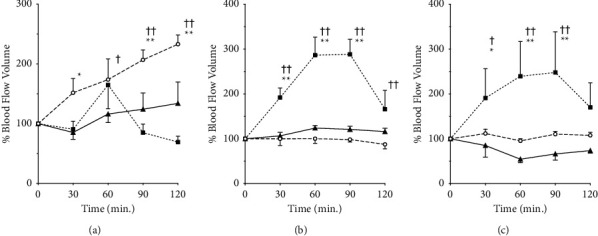
Effects of Gosha-jinki-gan and Keishi-bukuryo-gan on the blood flow volume. Changes in the blood flow volume of murine subcutaneous capillaries (a), arterioles (b), and arteries (c) before and after the administration of Gosha-jinki-gan (circle, *n* = 4), Keishi-bukuryo-gan (square, *n* = 3), and control (triangle, *n* = 3) are shown. All panels are expressed as percentages and those before administration as 100%. Each bar represents mean ± SEM. ^*∗*^: *P* < 0.05, ^*∗∗*^: *P* < 0.01 vs. control. ^†^: *P* < 0.05, ^††^: *P* < 0.01 vs. 0 min.

Figure 4 depicts the changes in the erythrocyte blood flow volume before and at 30–120 min after the administration of each Kampo prescription. In the capillaries, considering no change in the vessel diameter, the change in the blood flow rate followed a similar trend to that of the blood flow velocity; however, the amount of change was more pronounced. GJG increased the blood flow up to 232% of the pre-dose level in a sustained and progressive manner up to 120 min post-administration. In contrast, KBG increased the blood flow up to 60 min, and the change was insignificant (Figure 4(a)).

There were significant changes in the arterioles over time, with a significant increase in the blood flow by 286% of the pre-treatment level at 60 min following KBG administration (Figure 4(b)). We observed a significant change in the blood flow with a similar trend in the arteries; however, the increase was comparatively lower, with a maximum of 247% (Figure 4(c)). GJG did not cause significant changes in the arterial or arterial blood flow. The F- and *P* values of two-way ANOVA were as follows: *F*(8,32) = 4.4 and *P*=0.0011 for capillaries, *F*(8,28) = 14 and *P* < 0.0001, for arterioles and *F*(8,24) = 3.0 and *P*=0.0186 for arteries. The measured values of red blood cell velocity in each group before drug administration are provided in Additional Material 2. The imaging video files used for the analysis are provided in Additional Materials 3 (GJG) and 4 (KBG).

In the *oketsu* circulatory deficiency model, capillaries and arterioles are the target vessels of circulatory improvement by GJG by KBG, respectively.

Subsequently, we examined the effects of the two Kampo formulae on the rat *oketsu* model using the mesenterium.

**Figure 5:**
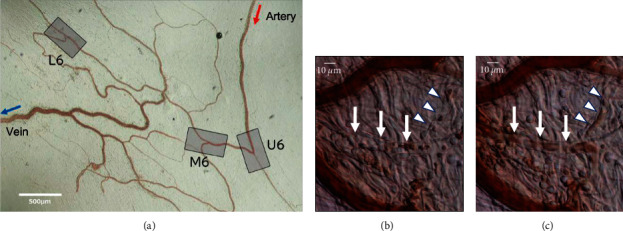
Typical live-imaging images of rat mesenterial regions. Representative images of a typically analyzed rat mesenterial region (a) Areas U6, M6, and L6 correspond to the upstream, midstream, and downstream of a mesenteric artery perfusion area, respectively. Panels (b) and (c) depict a series of representative images before and at 120 min after GJG administration in the mesenteric arterioles and capillaries. Before GJG administration Panel b, cell-free layers were extensively observed in the capillaries (arrow and arrowhead), whereas after GJG administration Panel c, erythrocytes were continuously observed in the vessels and cell-free layers were absent. The imaging video file used for the analysis is provided in Additional Material 5.

Figure 5(a) presents a typical image of the region analyzed from the mesenteric artery to the downstream capillaries. Figures 5(b) and 5(c) depict a series of representative images before and at 120 min after GJG administration in the mesenteric arterioles and capillaries. The original video of the image is provided in Additional Material 5. Before the administration of Kampo drugs, we observed erythrocyte congestion in the capillaries and the broadening of the cell-free layer, with the plasma layer lacking erythrocytes, with both circulatory deficiencies corresponding to typical *oketsu* status (Figure 5(b)). The circulatory deficiencies disappeared in the capillaries and arterioles following GJG and KBG administration, respectively (Figure 5(c)).

### 3.4. Effect of GJG on Microcirculation Is NO-Independent, While That of KBG Is NO-Dependent

**Figure 6:**
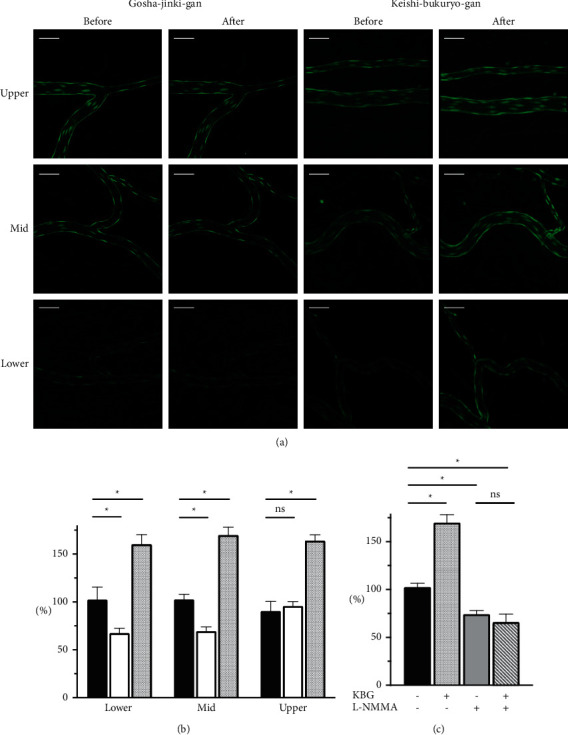
Changes in NO levels following the administration of Gosha-jinki-gan and Keishi-bukuryo-gan. Panel (a) depicts the representative DAF-2 fluorescence images of the upstream (upper panels), midstream (mid panels), and downstream (lower panels) of a mesenteric artery perfusion area, before and at 60 min after the administration of GJG (two rows on the left side) and KBG (two rows on the right side). The scale bar shown at the top of each panel corresponds to 50 *μ*m. Panel (b) depicts changes in fluorescence intensities 60 min after the administration of Kampo prescriptions. Black, white, and hashed bars indicate the control (*n* = 3), GJG (*n* = 6), and KBG (*n* = 3) groups, respectively. Lower, mid, and upper correspond to the upstream, midstream, and downstream of the mesenteric artery perfusion area, respectively. Panel (c) depicts changes in fluorescence intensities with or without KBG (*n* = 3 each) and an addition of NOS inhibitor L-NMMA. All panels are expressed as percentages and those before administration as 100%. Each bar represents the mean ± SEM. ^*∗*^: *P* < 0.05 vs. control, ns:.

Figure 6(a) presents typical fluorescence images of DAF-2 in the mesenteric vessels. Considering the constitutive NO production in the endothelium, we observed a certain amount of fluorescence in vascular endothelial cells without the administration of drugs.  Figure 6(b) depicts fluorescence changes in the endothelium after the second DAF-2DA perfusion. The fluorescence intensities are expressed as percentages and those before administration as 100%. Following GJG administration, DAF-2 fluorescence intensities were significantly decreased to 52.7% and 69.4% in the lower stream and midstream, respectively, compared to the first imaging in the control group (Figure 6(b)). In contrast, KBG significantly increased the fluorescence intensities in all regions (160% in the lower stream, 170% in the midstream, and 164% in the upper stream, Figure 6(b)). The inhibition of NOS with L-NMMA significantly decreased DAF-2 fluorescence and eliminated the effect of KBG (Figure 6(c)), indicating that the observed increase in DAF-2 fluorescence by KBG specifically generated NO.

## 4. Discussion

This study revealed that the target blood vessels of two types of herbal medicines, which are clinically known to increase blood flow but have different indications, differed according to their effects on NO. Antioxidative/nitrosative stress-related activity is one of the main pharmacological effects of herbal medicines, such as Kampo. Some of them display strong antioxidant activity *in vitro*, and others possess weak *in vitro* antioxidative activity while inducing endogenous *in vivo* antioxidant effects [18–21]. As per the previous studies, both KBG and GJG belong to the second category [3, 22].

KBG is typically prescribed for those with a firm constitution, ruddy face, and pain on either side of the navel on palpation, and with symptoms of *oketsu*, i.e., hot flashes, chillness in leg, and neck and shoulder stiffness [23]. KBG improves blood flow in both animal models and human [24–26], which mediates oxidative/nitrosative stress-related reactions [11, 27, 28]. We had previously reported the effect of KBG on blood flow using liveimaging [15]. In this study, we further developed the live-imaging method to identify the NO-producing sites following KBG and GJG administration. We found that KBG induced strong NO production from the vascular endothelium, mainly in the arterioles of the *oketsu* model (Figures 6(a) and 6(b)), together with vasodilation, and increased the blood flow in the subcutaneous arterioles and arteries. In contrast, NO fluorescence intensity was observed in the downstream region, which contained several capillaries. As capillary walls lack endothelial vascular smooth muscle cells, which are the target of NO-induced vasodilation, the increase in blood flow velocity in capillaries may not be attributed to the direct effect of NO on capillaries, rather to the increased blood flow in the upstream arterioles and improved erythrocyte aggregation.

Clinically, KBG is used in a *sho* termed “Jitsu-*sho*,” which denotes an excess pattern. Patients in the Jitsu-*sho* condition generally have excess energy and substantial muscle mass. Muscle changes in the blood flow due to cardiac output are regulated in the classical fast flow channel, i.e., the pathway from the arterioles to the small veins through the preferential capillaries [29, 30]. This pathway is consistent with the target vessels of KBG that we identified in this study. Therefore, the effect of improved arterial blood flow is more pronounced in patients with low muscle mass. Our results suggest that the traditional treatment decision for KBG, which considers the *sho* pattern, is based on NO dependency.

GJG is used in cases of fatigue, cold extremities, decreased urine output or polyuria, and occasionally thirst. Typical symptoms are leg pain, back pain, numbness, blurred vision in older adults, itching, dysuria, frequent urination, and swelling. In contrast to KBG, GJG increased the blood flow mainly in the capillary area, while this phenomenon in the *oketsu* model was NO-independent. Although the increasing effect of GJG on the peripheral blood flow is widely accepted [31–34], the underlying mechanism, particularly its relationship with NO, remains controversial. GJG has been suggested to increase the blood flow in an NO-dependent manner in the peripheral bloodstream of streptozotocin (STZ)-induced diabetic rats and the auricular peripheral arterioles and venules of STZ-treated mice [31, 34]. In addition, pharmacological effects of GJG related to NO increase have been reported in platelet aggregation, antinociceptive mechanism, and insulin resistance [35–37]. Aconiti tuber, a major crude component of GJG, reportedly increases the nitrite and nitrate levels in human plasma [38]. In contrast, an antioxidative but NO-independent blood flow-increasing effect was reported in an oxaliplatin-induced neurotoxicity rat model [18]. Hachimi-jio-gan is the original prescription of GJG and has a similar composition of crude drugs, except that it does not contain *Plantaginis semen* and *Achyranthis radix*. It reportedly suppresses inducible nitric oxide synthase (NOS) in animal models, such as chronic kidney disease and type 2 diabetes models, as well in acellular models [39–44]. Our findings in the *oketsu* model are contrary to those of reports claiming an increase in NO production by GJG.

One possible explanation is that we directly observed NO production from the vascular endothelium, whereas most of the abovementioned reports were guided by indirect observations, such as suppression experiments using NOS inhibitors, including NG-monomethyl-L-arginine. The *in vivo* metabolism of NO is complex, and NO reacts with multiple factors, including ROS (i.e., superoxide), thereby making direct *in situ* observation superior to assessments with metabolites. Furthermore, in the similar experimental system, KBG induced NO in the arteries and arterioles. Thus, we concluded that GJG does not increase NO production in the blood vessels under conditions of *oketsu* blood stasis. This is consistent with our findings that capillaries, which generally do not possess NOS-expressing cells, are the target vessels for the blood flow-improving effects of GJG observed in the subcutaneous vascular model.

In accordance with *sho*, GJG is frequently used for the kidney deficiency pattern (Jin-*kyo*: *kyo* of kidney). This is a pattern for insufficient amount of kidney qi, characterized by age-related symptoms, such as heaviness of the back or the lower legs, tinnitus or hearing loss, loss of hair or teeth, or sexual dysfunctions. Consequently, patients with this *sho* have relatively low muscle strength and physiological functions. This *sho* is characterized by low muscle mass and severe atherosclerosis than that in case of KBG. In contrast, the sustained increase in capillary blood flow may effectively improve the pathophysiological pattern (Figure 4(a)). Therefore, the NO-independent blood flow effect of GJG may be the mechanism behind the *sho*-based choice of treatment in traditional Kampo medicine.

## 5. Conclusions

Our study revealed that capillaries were the target vessels of GJG, and the time to exhibit the effect was slow and NO-independent. In contrast, KBG targeted the arterioles and arteries, and its effect was rapid and NO-dependent. To the best of our knowledge, this is the first study to analyze the pharmacological mechanisms of GJG and KBG and provide a modern scientific rationale for the traditional choice of prescription in Kampo and traditional herbal medicines.

## Data Availability

Part of the video data used to support the findings of this study are included within the supplementary information files as Additional Materials 3, 4, and 5. The other video data used to support the findings of this study were supplied by Timelapse Vision Co. Ltd. under license and so cannot be made freely available. Requests for access to these data should be made to the corresponding author.

## Supplementary Materials

Supplementary Materials Additional Material 2 Title: Vessel diameter, red blood cell blood flow velocity, and blood flow rate in each group before drug administration Description: Data are expressed as mean ± SEM. There were no significant differences among the three groups in the vessel diameter, red blood cell blood flow velocity, and blood flow rate before drug administration. Readers can access the mp4 files of Additional Materials 3 and 4 (and also 5) via the link address described in the manuscript (https://drive.google.com/drive/folders/1K6uikjdaT2Pf2Qjxp0ysXQq_hRgn2XGk?usp=sharing), (https://drive.google.com/drive/folders/1K6uikjdaT2Pf2Qjxp0ysXQq_hRgn2XGk?; deleted the last “usp=sharing”).
