# Supplementary material for: Live-Imaging Analysis of Target Vessels and Nitric Oxide Production Associated with Gosha-Jinki-Gan and Keishi-Bukuryo-Gan: Two Herbal Preparations with Clinically Proven Blood Flow-Improving Effects but with Different Traditional Clinical Indicative Patterns
Source: Evid Based Complement Alternat Med. 2022 May 11;2022:3821345. doi: 10.1155/2022/3821345 (PMC9117033; doi:10.1155/2022/3821345)
Supplement: Supplementary Materials — Additional Material 2 Title: Vessel diameter, red blood cell blood flow velocity, and blood flow rate in each group before drug administration Description: Data are expressed as mean ± SEM. There were no significant differences among the three groups in the vessel diameter, red blood cell blood flow velocity, and blood flow rate before drug administration. Readers can access the mp4 files of Additional Materials 3 and 4 (and also 5) via the link address described in the manuscript (https://drive.google.com/drive/folders/1K6uikjdaT2Pf2Qjxp0ysXQq_hRgn2XGk?usp=sharing), (https://drive.google.com/drive/folders/1K6uikjdaT2Pf2Qjxp0ysXQq_hRgn2XGk?; deleted the last “usp=sharing”). [file 3821345.f1.zip › 3821345.f1/Additional Material 2.pdf]

Additional Material 2

		Control	GJG		KBG	
		(mean±S.E.)	(mean±S.E.)	*P*	(mean±S.E.)	*P*
Diameter (μm)	Artery	61.6 ± 11.3	82.7 ± 4.7	0.125	76.4 ± 5.4	0.286
	Arteriole	39.0 ± 0.4	43.5 ± 0.4	0.375	45.0 ± 2.7	0.946
	Capillary	4.0 ± 0.1	4.1 ± 0.1	0.979	4.4 ± 0.2	0.722
Blood flow velocity (μm/sec)	Artery	$(8.56 \pm 1.58)x10^3$	$(5.77 \pm 0.99)x10^3$	0.241	$(6.89 \pm 0.84)x10^3$	0.525
	Arteriole	$(4.82 \pm 1.35)x10^3$	$(2.30 \pm 0.31)x10^3$	0.104	$(4.34 \pm 0.74)x10^3$	0.892
	Capillary	85.7 ± 12.1	74.2 ± 19.1	0.856	57.1 ± 18.7	0.484
Blood flow volume (μ m3/sec)	Artery	$(2.50 \pm 0.65)x10^7$	$(3.22 \pm 0.79)x10^7$	0.747	$(3.61 \pm 0.88)x10^7$	0.533
	Arteriole	$(3.97 \pm 1.22)x10^6$	$(3.77 \pm 1.00)x10^6$	0.987	$(4.95 \pm 1.06)x10^6$	0.775
	Capillary	$(11.16 \pm 2.22)x10^2$	$(9.21 \pm 1.78)x10^2$	0.791	$(8.87 \pm 3.44)x10^2$	0.756
